# Supplementary material for: Next-Generation CEA-CAR-NK-92 Cells against Solid Tumors: Overcoming Tumor Microenvironment Challenges in Colorectal Cancer
Source: Cancers (Basel). 2024 Jan 16;16(2):388. doi: 10.3390/cancers16020388 (PMC10814835; doi:10.3390/cancers16020388)
Supplement: Supplementary file 1 [file cancers-16-00388-s001.zip › cancers-2777952-supplementary.pdf]

Supplementary Material

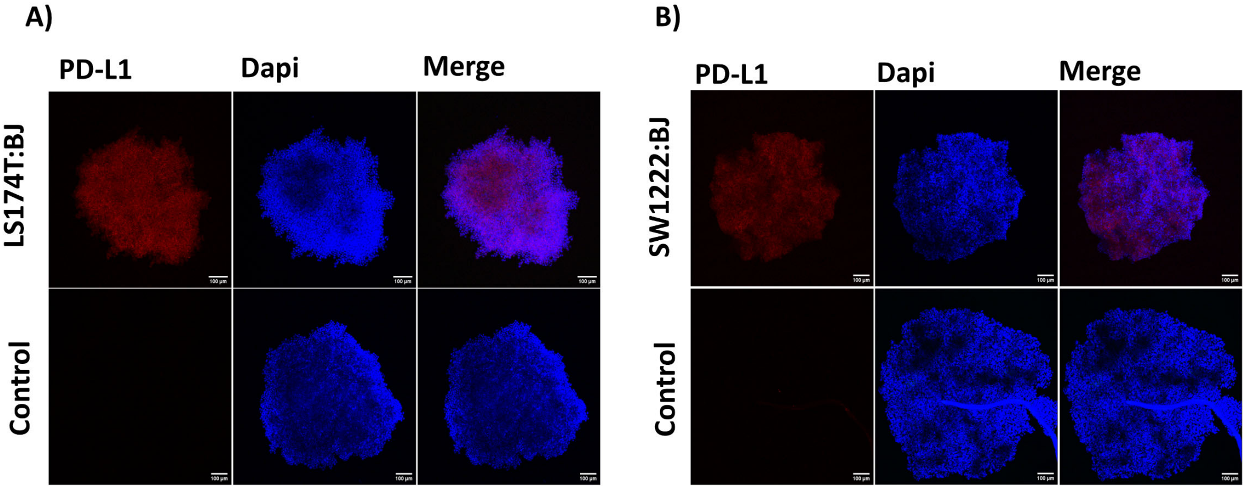

Figure S1. Co-culture tumor spheroids were stained with PD-L1 antibodies and imaged in a confocal microscope at x10 magnification. Isotype controls of the antibodies are shown directly beneath the measured sample spheroids. A) Images represent LS174T:BJ co-culture tumor spheroids stained for PD-L1 (red). An even dispersion of PD-L1 expression can be seen across the surface of the measured tumor spheroids B) Images represent SW1222:BJ co-culture tumor spheroids stained for PD-L1 (red). An even dispersion of PD-L1 expression can be seen across the surface of the measured tumor spheroids.
